# Solexa-Sequencing Based Transcriptome Study of Plaice Skin Phenotype in Rex Rabbits (*Oryctolagus cuniculus*)

**DOI:** 10.1371/journal.pone.0124583

**Published:** 2015-05-08

**Authors:** Lei Pan, Yan Liu, Qiang Wei, Chenwen Xiao, Quanan Ji, Guolian Bao, Xinsheng Wu

**Affiliations:** 1 Animal Husbandry and Veterinary Institute, Zhejiang Academy of Agricultural Sciences, Hangzhou, Zhejiang, China; 2 College of Animal Science and Technology, Yangzhou Uuniversity, Yangzhou, Jiangsu, China; 3 Chemistry and Life Science, Zhejiang Normal University, Jinhua, Zhejiang, China; The University of Tokyo, JAPAN

## Abstract

**Background:**

Fur is an important genetically-determined characteristic of domestic rabbits; rabbit furs are of great economic value. We used the Solexa sequencing technology to assess gene expression in skin tissues from full-sib Rex rabbits of different phenotypes in order to explore the molecular mechanisms associated with fur determination.

**Methodology/Principal Findings:**

Transcriptome analysis included *de novo* assembly, gene function identification, and gene function classification and enrichment. We obtained 74,032,912 and 71,126,891 short reads of 100 nt, which were assembled into 377,618 unique sequences by Trinity strategy (N50=680 nt). Based on BLAST results with known proteins, 50,228 sequences were identified at a cut-off E-value ≥ 10^-5^. Using Blast to Gene Ontology (GO), Clusters of Orthologous Groups (KOG) and Kyoto Encyclopedia of Genes and Genomes (KEGG), we obtained several genes with important protein functions. A total of 308 differentially expressed genes were obtained by transcriptome analysis of plaice and un-plaice phenotype animals; 209 additional differentially expressed genes were not found in any database. These genes included 49 that were only expressed in plaice skin rabbits. The novel genes may play important roles during skin growth and development. In addition, 99 known differentially expressed genes were assigned to PI3K-Akt signaling, focal adhesion, and ECM-receptor interactin, among others. Growth factors play a role in skin growth and development by regulating these signaling pathways. We confirmed the altered expression levels of seven target genes by qRT-PCR. And chosen a key gene for SNP to found the differentially between plaice and un-plaice phenotypes rabbit.

**Conclusions/Significance:**

The rabbit transcriptome profiling data provide new insights in understanding the molecular mechanisms underlying rabbit skin growth and development.

## Introduction

Rex Rabbit furs are highly appreciated by consumers for their inherent beauty, lightness, softness, and good warmth retention properties. Our team found that the quality of rabbit fur is affected by many factors, and its size is one of the significant standards for evaluation. This requires cultivation of large size populations in production. In recent years, some rex rabbits have shown wrinkles in abdomen and extremities during production; this phenotype is known as plaice. The skin size of wrinkle rabbits was found to be 15% larger than that of un-wrinkle animals, with the same quality fur. All the two phenotype rabbits are bred under identical condition:same forage, same temprature, and others. So it is probability that wrinkle phenotype was determined by same genes.

Rabbit skin development has not been widely studied and reports assessing wrinkle rabbits are scarce. Transcriptome analysis constitutes a starting point for studies of gene function and structure. Indeed, transcriptional profiling is a powerful approach for identification of genes globally and functionally expressed in various tissues[1] including skin. *De novo*[2–4] assembly of RNA-Seq data allows transcriptome analysis without the need for genome sequence[2]. In addition, transcriptome profiling has accelerated the discovery of new genes, providing a foundation for further research and molecular breeding.

Mammalian skin is composed of epidermis, dermis and subcutaneous tissue. The epidermal basal layer is formed through horny cell proliferation, differentiation and formation of the stratum corneum, eventually falling to achieve self-healing and renewal from the skin surface. The skin formation and regeneration is a complex three-dimensional process which results from regulation by signal factors from mesodermal tissues.

## Materials and Methods

### Ethics Statement

All animal experiments were reviewed and approved by the Institutional Animal Care and Use Committee of School of Animal Science and Technology, Yangzhou University and performed in accordance with the Regulations for the Administration of Affairs Concerning Experimental Animals (China,1988) and the Standards for the administration of experimental practices (Jiangsu, China, 2008). All surgery was performed according to recommendations proposed by European Commission (1997), and all efforts were made to minimize suffering of animals.

### Materials and Sample Preparation

The rex rabbit were selected from Zhejiang Yuyao Xinnong Rabbit Co., Ltd and raised according to the farm. During our experiment, rabbits were fed with pellet feed and green grass. Two 3-month-old full-sib Rex Rabbits each with plaice and un-plaice phenotypes, respectively, were evaluated. The animals were assigned numbers 7 and 728 (plaice, experiment group) and 9 and 724 (un-plaice, control group).

For anesthesia, 0.7% pentobarbital sodium respectively through ear vein injection at the dose of 6 mL·kg-1. The frist third dose of Pentobarbital sodium was injected within 5 min in order to mark rex rabbit into shallow sleep. And last two thirds of the dose were completed between 7 min to 13 min for make rex rabbit into deep sleep. The onset time of 0.7% pentobarbital sodium was 2–3 min, maintenance time was about 2 hours, the effect of muscle relaxants were well in anesthesia without inhibitory on the respiratory, and the anesthesia mortality rate was low. After anesthesia, a 1cm^2^ skin tissue sample was obtained from the buttock of the animals, placed immediately in liquid nitrogen, and preserved at -70°C until use. An iodine solution was smeared on the resultant lesion to prevent bacterial infection.

The skin samples with a size of approximately 1 cm^2^ were taken from the plaice and un-plaice parts on the buttock of each rabbit in Yangzhou University. From each sample pair, one was used for RNA extraction and the other stored at -70°C.

### cDNA Library preparation and Solexa sequencing

Total RNA was extracted using Trizol reagent following the manufacturer’s protocol(Invitrogen, USA). The RNA purity was evaluated by the ratio of OD_260_/OD_280_; RNA integrity number (RIN) value and RNA integrity was assessed by gel electrophoresis. RNA samples with OD_260_/OD_280_ ratio > 1.8 and RIN value > 8.0 were selected for deep sequencing. The samples for transcriptome analysis were prepared using Illumina’s kits following manufacturer’s recommendations. The cDNA library was sequenced on the Illumina HiSeq TM 2000.

### Unigene assembly and functional annotation

Raw reads obtained using Solexa RNA sequencing were cleaned by removing adaptors and low quality reads before assembly (Table 1) to obtain clean reads. Trinity[5] was used to assemble clean reads by paired-end. Unigenes[6] were identified until sequences can’t be extended, and used for Blastx search and annotation against databases (nr, Swiss-Prot, KEGG and KOG) a E-value cut-off of 10^-5^. The best aligned results were used to determine sequence direction of the unigenes. Functional annotation by Gene Ontology terms was analyzed by Blast2GO (http://www.blast2go.com) software[7]. The Blast2GO was used with default settings to assign GO(http://www.geneontology.org/) terms and enzyme codes to the predicted proteins based on their alignments to Swiss-Prot[8]. The KOG was used to classify orthologous gene products. Every protein in KOG was assumed to have evolved from an ancestor protein. This database contains putative protein sequences encoded by genes from bacteria, archaea and eukaryotes with complete genome sequences, and explores the evolutionary relationships between these groups through sequence analysis. Newly identified sequences can be aligned to the KOG database to predict and classify their possible functions[9].

**Table 1:** Reads before and after quality control and data validation.

Sample	Raw Data		Valid Data			Valid Ratio (Base)
	Read	Base	Read	Base	Average length	
Sample_7	73231128	7396343928	72686438	7261573180	99.90	98.18%
Sample_724	69371956	7006567556	68919754	6895336036	100.05	98.41%
Sample_728	74834696	7558304296	74227550	7416334363	99.91	98.12%
Sample_9	72881826	7361064426	72341822	7227307973	99.90	98.18%

### Identification of differentially expressed genes (DEGs) and pathway analysis

To identify the genes with significantly different expression levels between plaice and un-plaice phenotype rabbits, genes levels were derived using the FPKM method which is similarity to the method of RPKM[10]. FPKM taking into account the effect of depth and gene sequencing of fragments length count, is the most commonly used method to estimate the level of gene expression. P<0.05 and log_2_|FoldChange|>1 were used in the analysis. DEGs were mapped to each GO term and the numbers for each GO term were calculated. With Pathway enrichment, the main biochemical metabolic pathways and signal transduction pathways of DEGs were determined; pathways were based on the KEGG[11] pathway(http://www.genome.jp/kegg/) unit; KAAS (http://www.genome.jp/tools/kaas/) was used to predict the corresponding KO number. Then, genes and enzymes in KEGG annotation were analyzed and mapped to pathway information by KO number.

### qRT-PCR Validation

Five known skin development genes were selected from the DEGs for validation by qRT-PCR analysis. Each RT reaction consisted of 2.5 μg RNA, 2 μL 5×gDNA buffer with ddH2O added to 10 μL. The reaction was incubated at 42°C for 3 min. Then, 2 μL 10×Fast RT Buffer, 1 μL RT Enzyme Mix, 2μL FQ-RT Primer Mix, and 5 μL ddH2O were added to the 10 μL mixture, followed by incubation at 42°C for 15 min and 95°C for 3 min, to yield cDNA.

qRT-PCR was performed on a 7500 Real-Time PCR System(Applied Biosystems). The 20 μl PCR reaction mixture included 2 μL cDNA, 10 μL AceQTM qPCR SYBR Green Master Mix, 0.4 μL 50×ROX Reference Dye 2 (Vazyme), 0.4 μL forward primer, 0.4 μL reverse primer, and 6.8 μL ddH2O. Reactions were performed in a 96-well optical plate with the procedure described in Table 2. Samples were analyzed in triplicate. At the end of the PCR, melting curves were obtained at Stage 3 and analyzed to validate the specific generation of the expected PCR products. The expression levels of mRNAs were normalized to the glyceraldehyde 3-phosphate dehydrogenase(GAPDH) gene and calculated using the 2^-ΔΔCt^ method[12].

**Table 2:** qRT-PCR program details.

procedures	cycles	Temperature(°C)	Time
Stage 1	1	95	5 min
Stage 2	40	95	10 s
		60	34 s
Stage 3	1	95	15 s
		60	60 s
		95	15 s

### SNP test for target gene *lamb3*

Tanscriptome sequencing allows testing of individual SNPs to detect sequence differences among individuals or populations. SNP testing plays an important role in animal husbandry. By controlling or inducing the point mutation of some genes, SNP controls the biological phenotype. In our study, chosen target gene lamb3 for SNP testing and used Polymrphism Information Content(PIC) and χ^2^ to express the SNP. The formula of PIC is:

$$PIC=1-\sum_{i=1}^{k}p_{{}^{\;i}}{}^{2}-\sum_{i=1}^{k-1}\sum_{j=i+1}^{k}2p_{{}^{i}}{}^{2}p_{j}{}^{2}=2\sum_{i=1}^{k-1}\sum_{j=i+1}^{k}p_{i}p_{j}(1-p_{i}p_{j})$$

68 plaice and 108 un-plaice rex rabbit ear samples were obtained. The method anesthesia and minimize suffering after skin biopsy were same to the sample preparation of DNA. And DNA was extracted using Phenol-chloroform following the manufacturer’s protocol(TIANGEN, CHINA). The DNA purity was evaluated by the ratio of OD_260_/OD_280_; and DNA integrity was assessed by gel electrophoresis. DNA samples with OD_260_/OD_280_ ratio < 1.8.

The DNA was prepared for Signal-strand conformational polymorphism(PCR-SSCP).

## Results

### Assembly by unigenes

**Fig 1:**
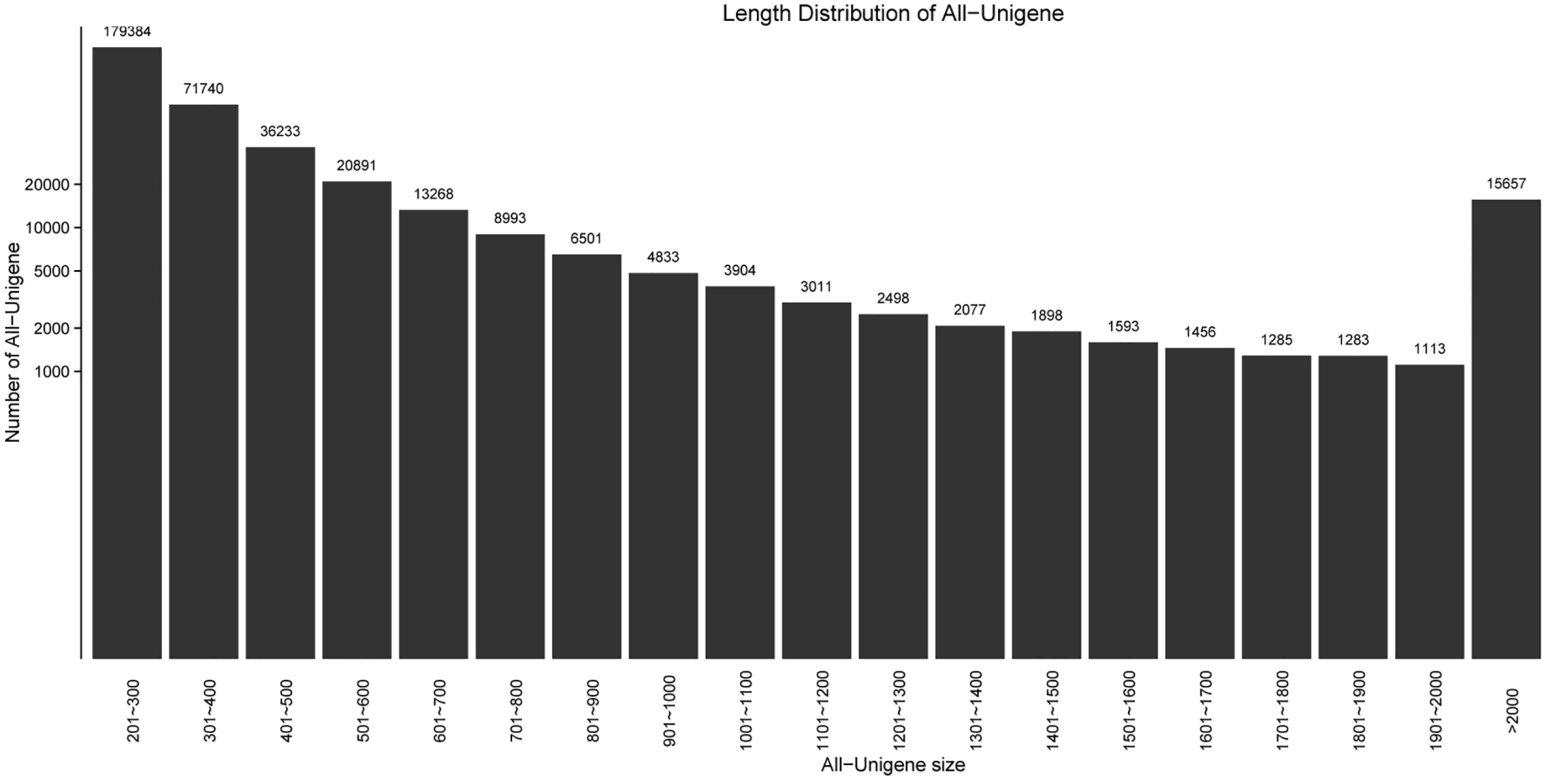
Length Distribution of all unigenes. The x-axis indicates sequence sizes from 200 nt to ≥ 2000 nt; the y-axis indicates the number of unigenes for a given sequence length.

A total of 377,618 unigenes were obtained, of which 287,357 (76.097%) and 54,486 (14.429%) were distributed in 200–500 nt and 500–1000 nt, respectively. There were 15,657 (4.146%) unigenes with more than 2,000 nt (Fig 1) and N50 was 680 nt. The longest unigene sequenced was 20,293 nt; 124 unigenes were longest than 10,000 nt. The raw data from Illumina deep-sequencing were deposited in the NCBI Short Read Archive (SRA) database with Accession number: PRJNA 271624. At present, there are no standard criteria to evaluate the quality of transcriptome assemblies[13]. Therefore, researchers assess the assembly quality mostly by evaluating its contiguity and accuracy[14]. However, unigene length is an important determinant of assembly quality: the longer the unigene, the better the contiguity.

### Functional classification of unigenes

A total of 377,618 unigenes were obtained in this study. Blast to NR, Swiss-prot, KOG, and KEGG database provided the unigene characteristics (Table 3). With GO analysis, the functions of these unigenes were determined. However, 85.5931% unigenes were not identified in any database, because the rabbit genomic information was not found. The Annotation for unigenes was shown in Table A in S1 File.

**Table 3:** All-in-one list of annotation.

Annotation database	No. of annotation	Percent of annotation (%)
**Total unigenes**	377,618	100
**NR**	50,228	13.3010
**Swiss-prot**	48,160	12.7536
**KOG**	60,593	16.0461
**KEGG**	13,431	3.5568
**GO**	42,895	11.3591
**Unknown**	323,215	85.5931

**Fig 2:**
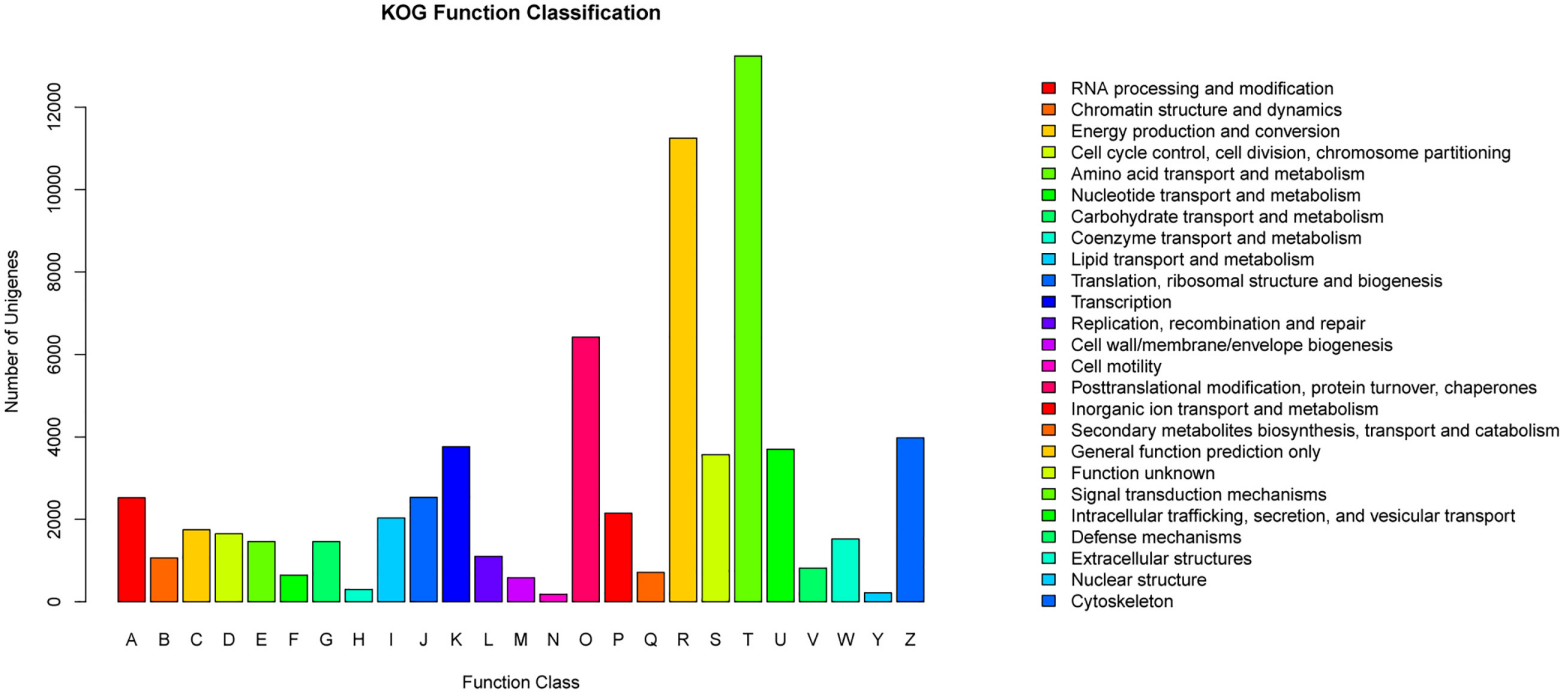
Histogram presentation of KOG classification. 60,593 sequences were grouped into 25 KOG categories. The information of right one-to-one correspond to every bar of left histogram.

**Fig 3:**
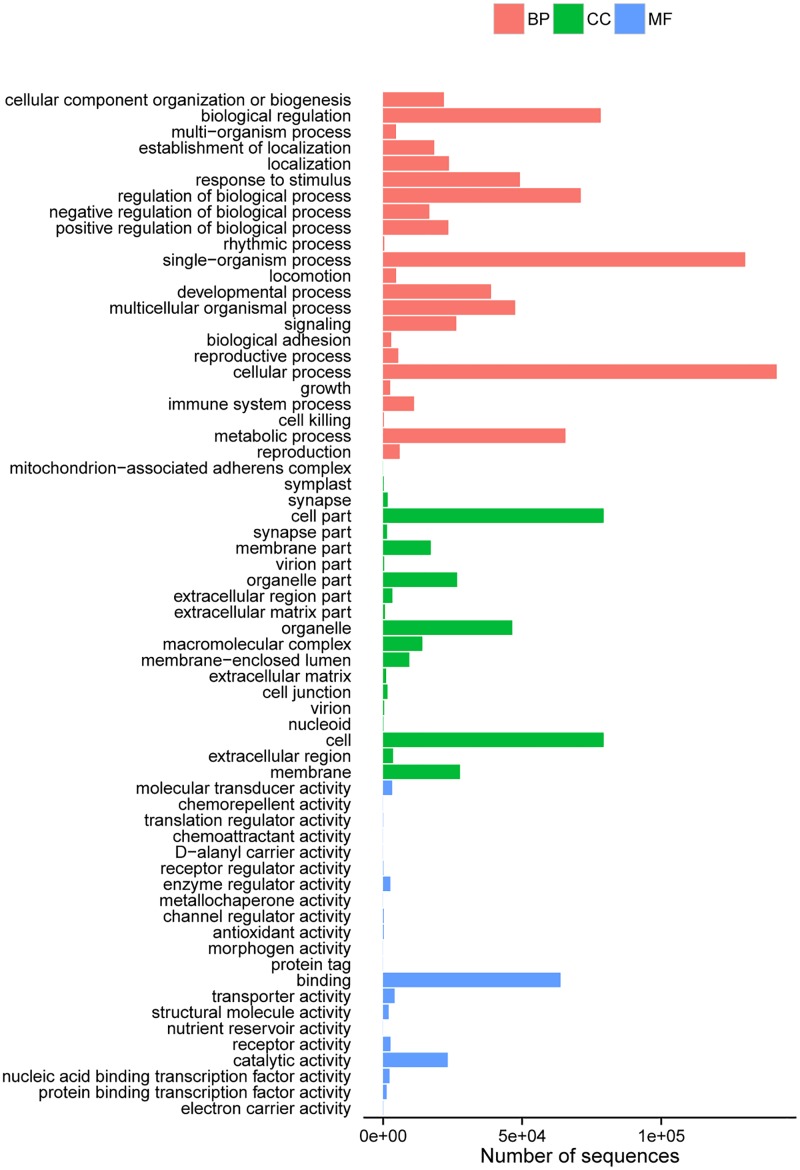
Histogram presentation of Gene Ontology classification. The results are summarized in three main categories: biological process, cellular component and molecular function. The y-axis on the right indicates the number of genes in a category. The y-axis on the left indicates the percentage of a specific category of genes in that main category.

KOG analysis classified 60,593 unigenes into 25 categories. From these, 13,240 unigenes were mapped to signal transduction mechanisms; 11,248 unigenes were attributed to general function prediction only; only 185 and 216 were classified into cell motility and nuclear structure, respectively (Fig 2). A total of 42,895 unigenes were blasted to different GO terms, and WEGO[15](http://wego.genomics.org.cn/cgi-bin/wego/index.pl)assigned the annotated genes into three levels, including Biological Process(BP), Cellular Component(CC) and Molecular Function(MF). With GO description and classification most genes were mapped into cellular process(GO:0009987), and single-organism process(GO:0044710) was followed (Fig 3).

### DEGs in rabbit skin

**Fig 4:**
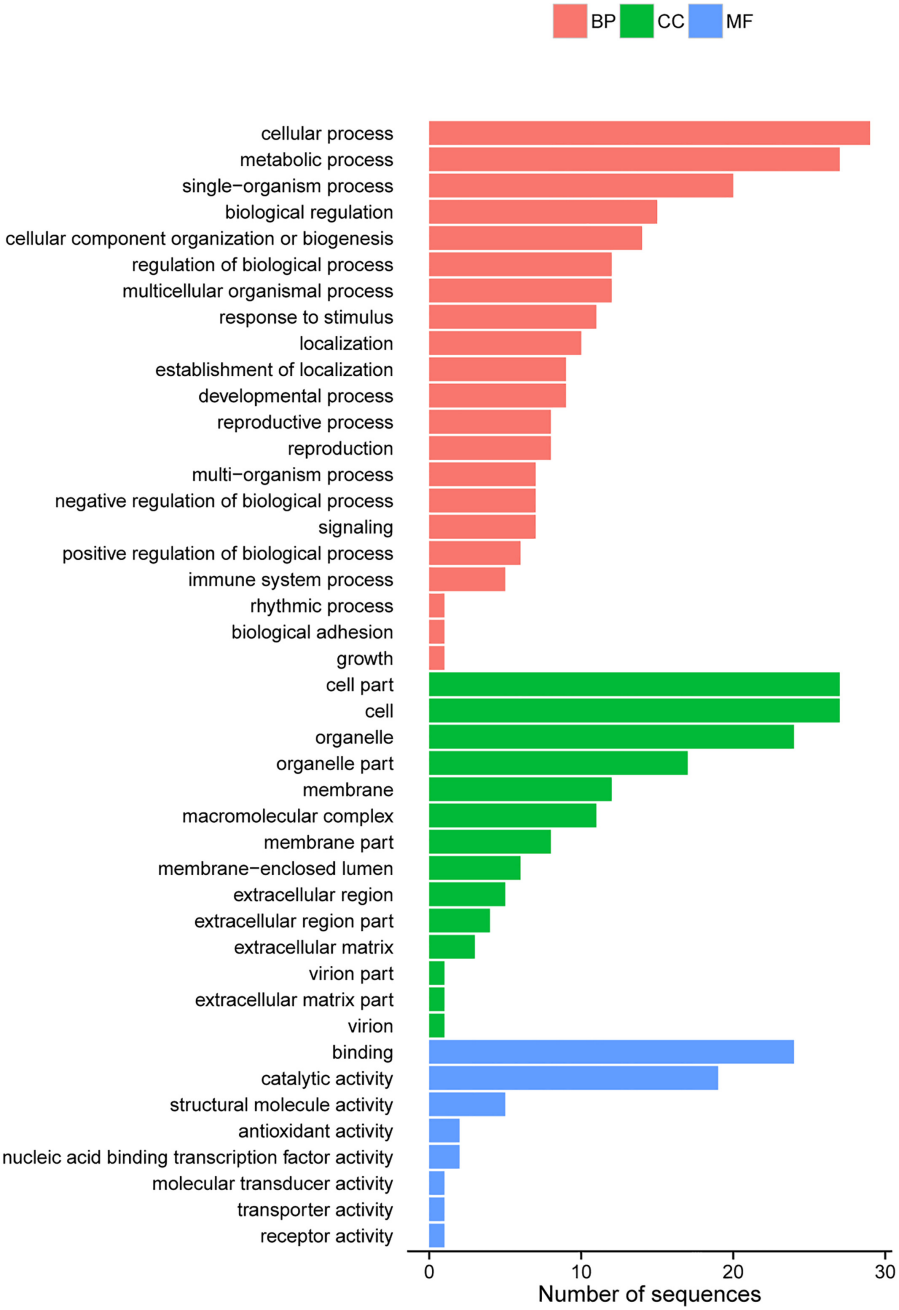
Histogram presentation of diff-Gene Ontology classification. The results are summarized in three main categories: biological process, cellular component and molecular function. The y-axis on the right indicates the number of genes in a category. The y-axis on the left indicates the percentage of a specific category of genes in that main category.

Using an algorithm based on a previously described method[16], genes differentially expressed between plaice and un-plaice phenotype rabbit skins were identified (P<0.05, log2|foldchange|>1). Comparing samples 7 and 9, we obtained 5,397 unigenes, including 2,105 and 3,292 that were up-regulated and down-regulated, respectively. The comparison between samples 728 and 724 yielded 6,191 unigenes, with 3,446 genes up-regulated and 2,745 genes down-regulated. When both gene groups were analyzed, 308 genes were obtained in both samples 7 and 9 on the one hand, and samples 728 and 724 on the other hand. Of the 308 genes, 118 were up-regulated, while 190 were down-regulated. However, only 99 of these genes were recognized by the GO software (Fig 4). The GO information about 99 known genes was shown in Table A in S2 File.

Therefore, the remaining 209 differentially expressed genes were considered novel. Of these, 75 and 134 were up-regulated (foldchange ≥ 2) and down-regulated (foldchange ≤ 0.5), respectively, in the skin samples from plaice rabbits compared with un-plaice animals. Interestingly, 33 novel genes were exclusively expressed in un-plaice samples, while 56 were only found in plaice samples(FPKM = 0.001). A total of 32 genes showed a log_2_|foldchange| > 3 in plaice versus un-plaice skin. The expression of differential expressed novel genes(four sheets) were shown in Table B in S2 File.

### KEGG pathway analysis and qRT-PCR

**Fig 5:**
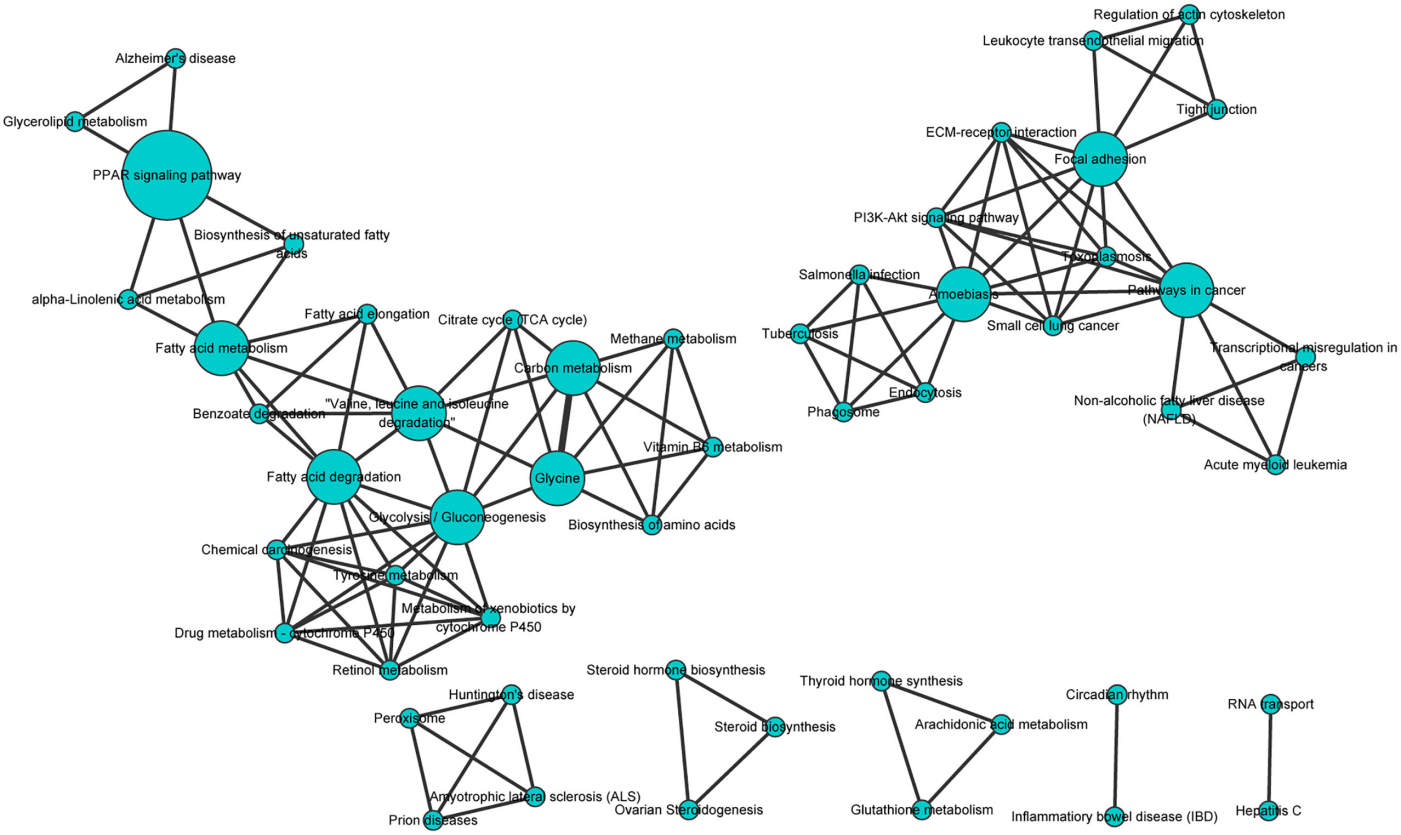
KEGG map of differentially expressed genes by the Cytoscape software. The size of circle indicates the number of unigenes, the line thickness indicates the number of overlapping unigenes.

The annotated sequences were mapped to the reference canonical pathways in KEGG[17]. A total of 13,431 sequences were assigned to 339 KEGG pathways, with most represented by unique sequences; PI3K-Akt(ko04151) signaling pathway was represented by 686(5.108%) sequences, whereas 683(5.085%) sequences were attributed to pathways in cancer(ko05200). The pathway enrichment of genes was shown in Table C in S2 File. For the 99 known genes, 29 were shown to be involved in PI3K-Akt signaling pathway, focal adhesion, ribosome, and PPAR signaling pathway, among others (Fig 5). KEGG pathways of differentially expressed genes were shown in Table D in S2 File.

**Fig 6:**
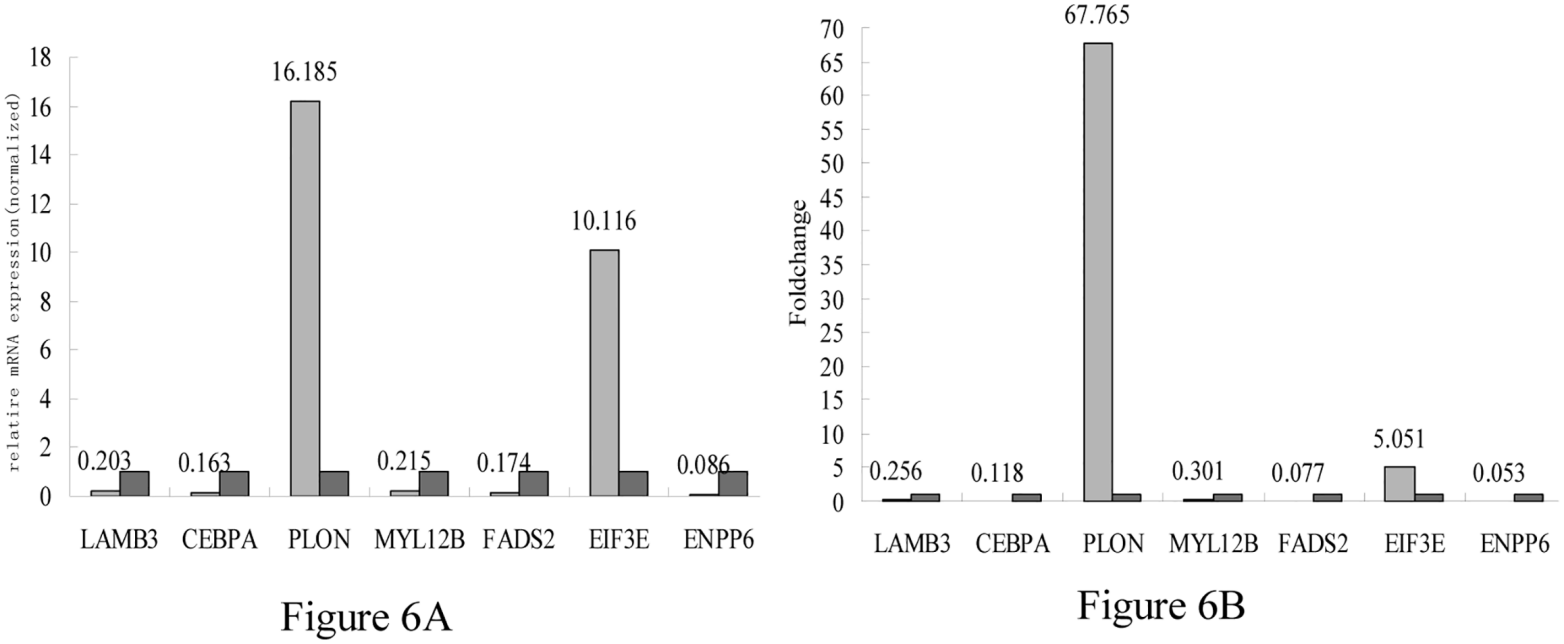
Analysis of the differentially expressed genes during skin growth and development in rabbits. (A), real time PCR validation of differentially expressed genes in plaice and un-plaice rabbit skin samples. (B), gene expression levels in plaice and un-plaice rabbit skin samples expressed as fold change. The red columns represent the control gene used for normalization (fold change set at 1).

Finally, seven target genes were selected to design specific primers (Table 4) for validation by qRT-PCR. Templates were obtained from the six nested groups of the two Phenotypes. In RNA-seq, *PLON* and *EIF3E* were up-regulated while *MYL12B*, *FADS2*, *LAMB3*, *CEBPA* and *ENPP6* were down-regulated. Similar results were obtained by qRT-PCR (Fig 6A and 6B).

**Table 4:** Primers used in qRT-PCR for the validation of differentially expressed genes.

Genes for qRT-PCR	qRT-PCR primer sequences
GAPDH	Forward Primer: 5’-TCACCATCTTCCAGGAGCGA-3’
	Reverse Primer: 5’-CACAATGCCGAAGTGGTCGT-3’
PLON	Forward Primer: 5’-CGTTCTAGTTGTTCAGTTC-3’
	Reverse Primer: 5’-AAAGATGTTCCTTGACTGC-3’
FADS2	Forward Primer: 5’-CATCCCTTTCTACGGCATC-3’
	Reverse Primer: 5’-GTAGGGTTCAAGGTCAATC-3’
ENPP6	Forward Primer: 5’-TCAACTTCGCCAATGCAGTC-3’
	Reverse Primer: 5’-ACTGGGTCATGTACTTCAGG-3’
MYL12B	Forward Primer: 5’-ACCAGTCACAGATTCAGGAG-3’
	Reverse Primer: 5’-CCTGGAGCCTCATTCATCA-3’
LAMB3	Forward Primer: 5’-GCGATTCCAGCAACTCGAAG-3’
	Reverse Primer: 5’-ACAGAAGCAGCTCCCATGCAG-3’
CEBPA	Forward Primer: 5’-GCTCGGGTAAGGCCAAGAA-3’
	Reverse Primer: 5’-AGGCGGTCATTGTCACTGG-3’
EIF3E	Forward Primer: 5’-GCGTTATTTGACCACAGCAG-3’
	Reverse Primer: 5’-AAGCACTGATTCACATTCCC-3’

*PLON* also called pol ν, is a newly discovered a family polymerase that generates a high error rate when incorporating nucleotides opposite dG. And pol ν is involved in homologous recombination in responds to various DNA cross-link repair[18]. *EIF3E*(translation initiation factor 3 subunit E) involved in the regulation of cell growth and cell cycle, and is closely related to cancer. EIF3E is the key factor for eIF4E to recruit the eIF4E kinase Mnk1(MAPK signal-integrating kinase 1) to eIF4F[19]. Myosin regulatory light chain and essential light chain compose the light chain of myosin. *MYL12B* is an importent chain of regulatory light chain in non-muscle cell. *FADS2*(fatty acid desaturase 2) catalyzes the first desaturation step, plays a vital role and thus was commonly used to indicate the LC-PUFA synthesis capacity[20]. *ENPP6*(ectonucleotide pyrophosphatase) is one of ENPP family. And *ENPP6* is able to hydrolyze LPC, GPC, SPC. *Lamb3* was blast to the epidermis development by GO. And as a transcription factors, C/EBPα play a key role in epidermis cuticular layer.

### SNP test for target gene *lamb3*

Reverse sequencing of PCR products showed 1 SNP site in the exon8 of *lamb3*. Respectively, the SNP was sense mutations. The Statistics (Table 5) showed that the Gene frequency of C and T in two phenotype samples were different and the χ^2^ also showed the plaice genetic traits do not meet the the Hardy-Weinberg Low in the growth and development of rabbit skin.

**Table 5:** Genetic diversity of eighth exon of *lamb3* in white Rex Rabbit.

Phenotype of rabbits	Samples	Genotype frequencies			Gene frequency		PIC	χ2
		CC	CT	TT	C	T		
Plaice rabbit	68	0.455 (1)	0.015 (44)	0.530 (23)	0.338	0.662	0.348	13.500
Un-plaice rabbit	108	0.482 (20)	0.049 (56)	0.469 (32)	0.444	0.556	0.372	0.2729

**Fig 7:**
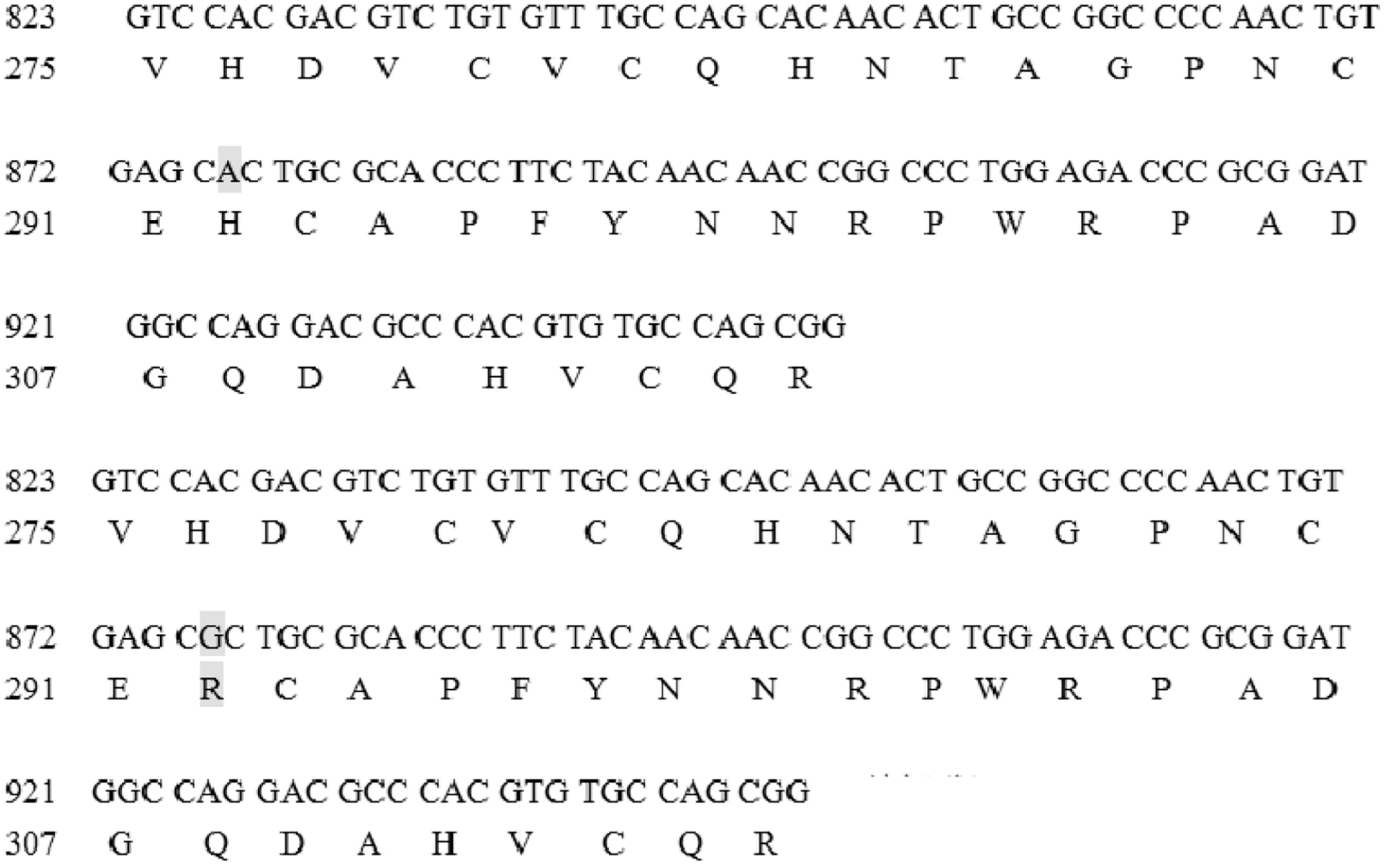
The nucleotide and amino acid sequences of eighth exon of lamb3. The upper one was the amino acid sequence of exon8 in LAMB3 in un-plaice rex rabbit; and the under one was the sequence in plaice rex rabbit.

In plaice rabbit we found that the 18th Amino Acids was Arginine but in un-plaice rabbit was Histidine (Fig 7). This mutations can alter the structure of protein, perhaps making it function differently.

## Discussion

The expression and functional analysis of growth and transcription factors in embryonic skin identified skin development as a complicated network and a highly regulated process. The major growth factors involved were determined to be EGF, FGF, KGF, TGF, IGF and PDGF. For instance, it has been reported that both skin development and phenotype maintenance require EGF [21–23]. In addition, EGF promotes keratinocyte migration and proliferation, and regulates the expression of K6 and K16[24]. Furthermore, EGF was shown to induce more epithelial stem cells to line along the stratum basale and form clusters (so-called stem cell island) in the granular and spinous layers [25–26]. These findings suggested that EGF activation of stem cells occurs not only in the stratum basale but also in other epidermal layers, and therefore could quickly increase the number of epithelial cells and epidermis thickness to enhance protection and accelerate the epidermal renewal [26]. Interestingly, EGF and EGFR were expressed in human skin tissues at different developmental stages, indicating that these growth factors play an important role in skin genesis, structural and functional maintenance, and recovery.

Transcription factors (TFs) perform important regulatory functions by controlling a variety of cell processes[27–28]. Through TF action, cell growth factors can better affect skin growth and development. CCAAT/enhancer binding proteins (C/EBPs) are distributed in epidermis cuticular layer[29]. Studies[30–31] have found that C/EBPα, C/EBPβ, and C/EBPδ participate in cutin cell differentiation and inhibition of growth. The GO and KEGG pathway analyses revealed that most DEGs were associated with cellular process and metabolic ontology categories. Of the DEGs, the *lamb3* gene was shown to be related to epidermis development. LAMB3 is a glycoprotein and an important component of basement membrane. In addition, LAMB3 plays an important role in the formation and stabilization of the basement membrane. Finally, lamb3 controls cell activity by interacting with cells directly or indirectly, and plays a role in cellular events such as adhesion or transfer, differentiation or polarization, proliferation or apoptosis, and gene expression[32]. For instance, *lamb3* is an important gene in the PI3K-Akt signaling pathway, focal adhesion and ribosome. PI3K-Akt signaling is a regulatory pathway involved in many biological functions. With the regulation of PI3K, Akt can modulate the activity of a variety of protein molecules to perform physiological responses such as cell survival and proliferation, angiogenesis, metabolic regulation and cell migration[33]. *lamb3* was also shown to be associated with human Junctional Epidermolysis Bullosa (JEB)[34]. JEB occurs in Zona pellucid of the dermo-epidermal basal membrane. The ultrastructure showed abnormal hemi-desmosome anchoring filament complex with *lamb3* involved in the synthesis of hemi-desmosome. The changes of LAMB3 protein many the key factor for differently skin phenotype in rex rabbit. In addition to regulating cell growth and migration, focal adhesion signaling pathway also plays a role in animal development throughout the process of embryonic development. Many tissues including skin require FAK for their development. *lamb3* and GF are simultaneously involved in the above two pathways and jointly affect skin development. In focal adhesion pathway, lamb3 and GF regulate FAK through their corresponding receptors, therefore modulating PI3K; in the PI3K-Akt signaling pathway, both *lamb3* and GF stimulate PI3K to participate in various signaling pathways. *lamb3* regulates GF expression by modulating the downstream gene expression, further controlling skin development. Meanwhile, CEBPA, lamb3 and GF were shown to jointly participate in pathways in cancer. CEBPA regulates skin development through indirect regulation of GF and *lamb3*.

We performed fluorescent quantitative PCR to validate the expression of selected candidate genes in both the plaice and un-plaice samples. qRT-PCR showed that *PLON* expression level was 16.185 times higher in plaice samples compared with un-plaice counterparts. Although a fold change of 67.765 was obtained for this gene in the transcriptome study, both results agree that *PLON* is up-regulated in plaice samples. The difference in fold change could be attributed to experimental errors either in qRT-PCR or sequencing process.

Through RNA sequencing, novel genes were found in Rex Rabbit skin tissues, with some of them differentially expressed in the plaice and un-plaice phenotypes. These genes were poorly annotated and further information could not be obtained by BLAST in NCBI (including EST); they might be Rex-Rabbit skin specific genes. The genes that were highly expressed in one phenotype but not the other (FPKM = 0.001) are likely to be critical growth factors or/and transcription factors in skin development.

## Conclusion

This study was the first high-throughput-sequencing-based transcriptome research assessing skin tissues of Rex Rabbits of different phenotypes. We found 29 known genes directly or indirectly associated with skin development in Rex Rabbits, including *lamb3*, which was the most directly linked gene. Moreover, 209 novel genes were discovered from the differential expression analysis. These genes, especially those specifically expressed in one phenotype but not the other, for example the 56 genes that were only expressed in Rex Rabbits with plaice phenotype, should be studied to further characterize skin development in domestic rabbits.

## Supporting Information

S1 File Table A, Annotation for unigenes.
(ZIP)

S2 File Table A, The GO information about 99 known genes. Table B, The expression of differential expressed novel genes (four sheets). Table C, The pathway enrichment of genes. Table D, KEGG pathways of differentially expressed genes.
(ZIP)
