# Supplementary material for: Solexa-Sequencing Based Transcriptome Study of Plaice Skin Phenotype in Rex Rabbits (Oryctolagus cuniculus)
Source: PLoS One. 2015 May 8;10(5):e0124583. doi: 10.1371/journal.pone.0124583 (PMC4425669; doi:10.1371/journal.pone.0124583)
Supplement: S2 File — Table A, The GO information about 99 known genes. Table B, The expression of differential expressed novel genes (four sheets). Table C, The pathway enrichment of genes. Table D, KEGG pathways of differentially expressed genes. (ZIP) [file pone.0124583.s002.zip › Table_C.docx]

Table S4 KEGG pathways of differentially expressed genes

| Pathway_ID | Pathway_Designation | diff-gene No. |
| --- | --- | --- |
| ko05321 | Inflammatory bowel disease (IBD) | 1 |
| ko05222 | Small cell lung cancer | 1 |
| ko05221 | Acute myeloid leukemia | 1 |
| ko05204 | Chemical carcinogenesis | 1 |
| ko05202 | Transcriptional misregulation in cancers | 1 |
| ko05200 | Pathways in cancer | 2 |
| ko05160 | Hepatitis C | 1 |
| ko05152 | Tuberculosis | 1 |
| ko05146 | Amoebiasis | 2 |
| ko05145 | Toxoplasmosis | 1 |
| ko05132 | Salmonella infection | 1 |
| ko05020 | Prion diseases | 1 |
| ko05016 | Huntington's disease | 1 |
| ko05014 | Amyotrophic lateral sclerosis (ALS) | 1 |
| ko05010 | Alzheimer's disease | 1 |
| ko04932 | Non-alcoholic fatty liver disease (NAFLD) | 1 |
| ko04918 | Thyroid hormone synthesis | 1 |
| ko04913 | Ovarian Steroidogenesis | 1 |
| ko04810 | Regulation of actin cytoskeleton | 1 |
| ko04710 | Circadian rhythm | 1 |
| ko04670 | Leukocyte transendothelial migration | 1 |
| ko04530 | Tight junction | 1 |
| ko04512 | ECM-receptor interaction | 1 |
| ko04510 | Focal adhesion | 2 |
| ko04151 | PI3K-Akt signaling pathway | 1 |
| ko04146 | Peroxisome | 1 |
| ko04145 | Phagosome | 1 |
| ko04144 | Endocytosis | 1 |
| ko03460 | Fanconi anemia pathway; | 1 |
| ko03320 | PPAR signaling pathway | 3 |
| ko03040 | Spliceosome | 1 |
| ko03030 | DNA replication | 1 |
| ko03013 | RNA transport;ko05160, Hepatitis C | 1 |
| ko03010 | Ribosome | 4 |
| ko01230 | Biosynthesis of amino acids | 1 |
| ko01212 | Fatty acid metabolism | 2 |
| ko01200 | Carbon metabolism | 2 |
| ko01040 | Biosynthesis of unsaturated fatty acids | 1 |
| ko00982 | Drug metabolism - cytochrome P450 | 1 |
| ko00980 | Metabolism of xenobiotics by cytochrome P450 | 1 |
| ko00900 | Terpenoid backbone biosynthesis | 1 |
| ko00830 | Retinol metabolism | 1 |
| ko00750 | Vitamin B6 metabolism | 1 |
| ko00680 | Methane metabolism | 1 |
| ko00620 | Pyruvate metabolism | 1 |
| ko00604 | Glycosphingolipid biosynthesis - ganglio series | 1 |
| ko00600 | Sphingolipid metabolism | 1 |
| ko00592 | alpha-Linolenic acid metabolism | 1 |
| ko00590 | Arachidonic acid metabolism | 1 |
| ko00565 | Ether lipid metabolism | 1 |
| ko00561 | Glycerolipid metabolism | 1 |
| ko00532 | Glycosaminoglycan biosynthesis - chondroitin sulfate / dermatan sulfate | 1 |
| ko00480 | Glutathione metabolism | 1 |
| ko00362 | Benzoate degradation | 1 |
| ko00350 | Tyrosine metabolism | 1 |
| ko00280 | Valine, leucine and isoleucine degradation | 2 |
| ko00260 | Glycine, serine and threonine metabolism | 2 |
| ko00240 | Pyrimidine metabolism | 1 |
| ko00140 | Steroid hormone biosynthesis | 1 |
| ko00100 | Steroid biosynthesis | 1 |
| ko00071 | Fatty acid degradation | 2 |
| ko00062 | Fatty acid elongation | 1 |
| ko00020 | Citrate cycle (TCA cycle) | 1 |
| ko00010 | Glycolysis / Gluconeogenesis | 1 |
